# Radiological findings in megaesophagus secondary to Chagas disease: chest X-ray and esophagogram*

**DOI:** 10.1590/0100-3984.2015.0141

**Published:** 2016

**Authors:** Thiago Giansante Abud, Lucas Giansante Abud, Vanessa Sales Vilar, Denis Szejnfeld, Samuel Reibscheid

**Affiliations:** 1 MsC, Doctoral Student in the Department of Diagnostic Imaging of the Escola Paulista de Medicina da Universidade Federal de São Paulo (EPM-Unifesp), Interventional Radiologist at the Hospital Israelita Albert Einstein, São Paulo, SP, Brazil.; 2 MD, Radiologist at Documenta - Hospital São Francisco, Doctoral Student in the Faculdade de Medicina de Ribeirão Preto da Universidade de São Paulo (FMRP-USP), Ribeirão Preto, SP, Brazil.; 3 PhD, Radiologist at Documenta - Hospital São Francisco, Ribeirão Preto, SP, Brazil.; 4 PhD, Interventional Radiologist, Department of Diagnostic Imaging of the Escola Paulista de Medicina da Universidade Federal de São Paulo (EPM-Unifesp), São Paulo, SP, Brazil.; 5 PhD, Radiologist, Department of Diagnostic Imaging of the Escola Paulista de Medicina da Universidade Federal de São Paulo (EPM-Unifesp), São Paulo, SP, Brazil.

**Keywords:** Esophageal achalasia/radiography, Esophagus/radiography, Radiography, thoracic

## Abstract

**Objective:**

To identify and classify the radiographic patterns of megaesophagus in Chagas disease, as seen on esophagograms and chest X-rays.

**Materials and Methods:**

This was a prospective study of 35 patients diagnosed with esophageal disease via manometry. The changes found on esophagograms were stratified according to Rezende's classification, divided into four categories (grades I through IV) determined by the degree of dilatation and impairement of esophageal motility. We subsequently correlated that ranking with the chest X-ray findings: gastric air bubble; air-fluid level; and mediastinal widening.

**Results:**

Among the 35 patients, the esophageal disease was classified as grade I in 9 (25.7%), grade II in 3 (8.6%), grade III in 19 (54.3%), and grade IV in 4 (11.4%). None of the patients with grade I esophageal disease showed changes on chest X-rays. In two of the three patients with grade II disease, there was no gastric air-bubble, although there were no other findings in any of the grade II patients. Of the 19 patients with grade III disease, 15 had abnormal findings on X-rays. All four patients with grade IV disease showed abnormalities.

**Conclusion:**

The use of Rezende's classification is feasible, encompassing findings ranging from the subtle changes that characterize the initial phases of esophageal disease to the complete akinesia seen in dolicomegaesophagus. Chest X-ray findings are more common in patients with advanced stages of the disease and indicate the degree of esophageal involvement in Chagas disease.

## Abstract (pt)

**Objetivo:**

Identificar e classificar as alterações radiológicas no megaesôfago chagásico no esofagograma e na radiografia simples de tórax.

**Materiais e Métodos:**

Foram estudados 35 pacientes com diagnóstico de esofagopatia na manometria. As alterações encontradas no esofagograma foram estratificadas segundo a classificação de Rezende, dividida em quatro categorias, determinadas pelo grau de dilatação e alteração da motilidade do esôfago. Também foi realizada correlação desta classificação com os achados na radiografia de tórax: presença ou ausência de bolha gástrica, nível líquido e alargamento do mediastino.

**Resultados:**

A distribuição encontrada, segundo a classificação de Rezende, foi: grau I - 25,7% (9/35); grau II - 8,6% (3/35); grau III - 54,3% (19/35); grau IV - 11,4% (4/35). Nenhum paciente grau I apresentou alterações na radiografia simples. No grau II, o único achado foi a ausência da bolha gástrica (2/3). No grau III, 15 dos 19 pacientes apresentaram achados anormais na radiografia. Já no grau IV, em todos os quatro pacientes identificaram-se anormalidades no exame simples.

**Conclusão:**

A classificação de Rezende é praticável, encontrando-se desde achados sutis caracterizando os graus iniciais até a completa acinesia do dolicomegaesôfago. Os achados na radiografia de tórax são mais frequentes em pacientes com estágios avançados da doença e podem fazer aventar o grau da esofagopatia chagásica.

## INTRODUCTION

According to the World Health Organization, there are currently 18-20 million people contaminated by the causative agent of Chagas disease. In Brazil, 5-6 million people are so infected, underscoring the importance of the disease in the country^(1,2)^.

Chagas disease is caused by the protozoan parasite *Trypanosoma cruzi*, which was first described by Carlos Chagas in 1909. Chagas characterized it as a human parasite, identifying it in the blood of a nine-month-old baby who developed an acute form of the disease that came to bear the name of the author. Chagas also described the life cycle of *T. cruzi* in the invertebrate *Triatoma infestans*, popularly known as the reduviid bug, or "kissing bug"^(1)^.

Achalasia in Chagas disease, caused by denervation of the nerve plexuses and immune response, can evolve to considerable dimensions, often showing visible signs on routine chest X-rays^(3)^.

Through barium contrast-enhanced imaging of the esophagus (barium swallow examination), the degree of esophageal involvement can be determined according to Rezende's classification. The barium swallow examination can identify the early stages of esophageal involvement by revealing subtle signs such as mild hypotonia and tertiary waves^(4)^.

The objectives of this study were to identify esophageal changes and classify the degree of esophageal involvement seen in contrast-enhanced images of the esophagus, according to Rezende's classification, as well as to identify changes on routine chest X-rays, correlating those changes with the degree of megaesophagus seen on barium swallow examinations, also according to Rezende's classification, in patients with Chagas disease referred to a radiology clinic for investigation of complaints of dysphagia.

## MATERIALS AND METHODS

This was a prospective study conducted between June 2003 and April 2004, involving 35 patients referred to our department of diagnostic imaging, all of whom tested positive for on the "Machado Guerreiro" battery of tests (indirect hemagglutination, indirect immunofluorescence, and enzyme-linked immunosorbent assay) and were diagnosed with megaesophagus (by barium swallow examination). Patient ages ranged from 25 to 76 years (mean, 58.27 years). Of the 35 patients evaluated, 23 were female and 12 were male.

## Chest X-ray

The chest X-ray technique employed was high kV and low mAs. In all cases, posteroanterior and lateral X-rays were obtained.

We evaluated the following changes on chest X-rays:

- Absence of the gastric air bubble: When there is functional stenosis of the gastric cardia, air ceases to be swallowed and thus the gastric air bubble ceases to be visualized. Although the lack of the gastric air bubble is a nonspecific X-ray finding, it is quite sensitive in cases in which there is a complaint of severe dysphagia.
- Mediastinal widening: Although mediastinal widening is most often right-sided and inferior (right paracardiac), it can also occur on the left side and at any level.
- Air-fluid level: Within the esophagus, stasis of food residue results in the formation of an air-fluid level, which can be seen on a chest X-ray.

## Barium swallow

The barium swallow examination involved oral administration of the contrast agent barium sulfate. An experienced radiologist evaluated the images in real time by fluoroscopy and took X-rays when necessary. Standardized X-rays were obtained in right anterior oblique, lateral, and anteroposterior views. To identify dilation, X-rays of the esophagus were taken.

During the barium swallow examination, the following changes were evaluated:

- Altered motility (defined as tertiary waves, hypokinesia, or akinesis).
- Gastric emptying velocity.
- Altered caliber of the esophagus, air-barium levels, and air-fluid levels.
- Presence of the "bird beak" sign.

**Figure 1:**
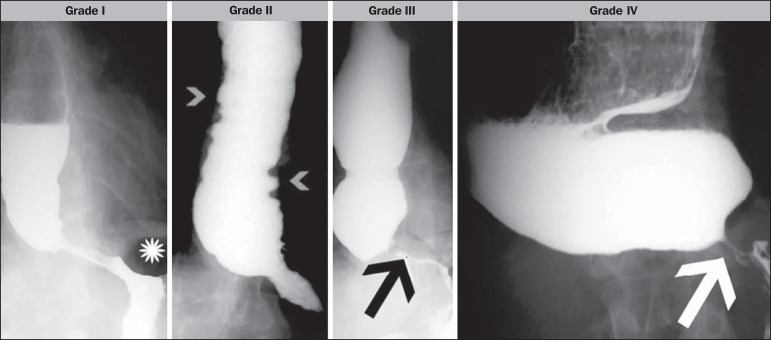
Rezende's classification. Grade I - esophageal hypotonia and gastric air bubble (asterisk). Grade II - moderately dilated esophagus and tertiary waves (arrowheads). Grade III - esophageal dilation and "bird beak" sign of the gastric cardia (arrow); tertiary waves are present, but less frequently. Grade IV - akinesis and dolicomegaesophagus, with the "bird beak" sign of the gastric cardia (arrow).

Esophageal involvement was graded according to Rezende's classification (Table 1 and Figure 1), and the chest X-ray findings were correlated with the results of the barium swallow examination^(4)^. The X-rays and barium swallow examinations were evaluated, in consensus, by two radiologists with 4 and 30 years of experience, respectively.

**Table 1:** Rezende’s classification^(4)^.

Grade I	The esophagus shows difficult emptying and mild hypotonia, with episodes of tertiary waves and no dilation.
Grade II	Contraction of the muscles of the gastric cardia (achalasia). The esophagus shows a mild to moderate increase in caliber; tertiary waves are more frequent.
Grade III	The esophagus shows an evident increase in caliber. The distal portion has the classic “bird beak” sign. The majority of cases with total akinesis of the esophagus show violent contractions of the circular musculature.
Grade IV	In addition to the changes described for grade III involvement, we observed intense dilation of the esophagus, which seems to rest on the right phrenic hemidiaphragm. We refer to this as severe (sigmoid) megaesophagus.

The data distribution was analyzed and the groups were compared.

## RESULTS

Among the 35 patients evaluated, the Rezende's classification was grade I in 9 (25.71%), grade II in 3 (8.57%), grade III in 19 (54.28%), and grade IV in 4 (11.42%).

Except for those classified as having grade IV esophageal disease, all of the patients showed tertiary waves during the dynamic evaluation of esophageal motility in the barium swallow examination.

**Figure 2:**
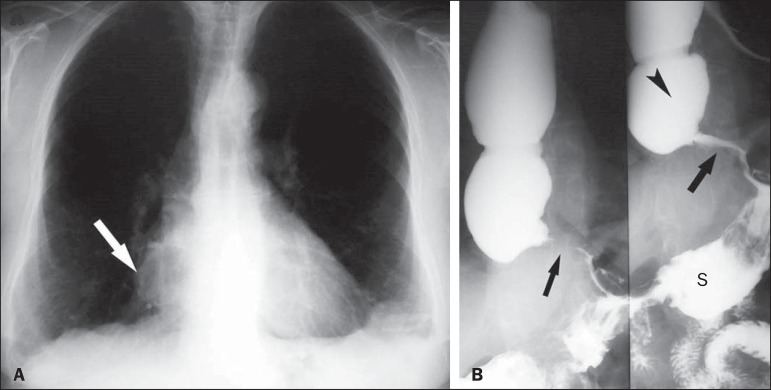
Patient with grade III megaesophagus. **A**: Posteroanterior, double-contrast X-ray image of the right lower arch (arrow). **B**: Barium swallow examination showing esophageal dilation (arrowhead) and the typical "rat-tail" sign in the distal portion of the esophagus (arrows). During the test, air passes into the stomach, forming the gastric air bubble, and the stomach (S) has a normal appearance. Discrete tertiary waves are observed during the examination.

**Figure 3:**
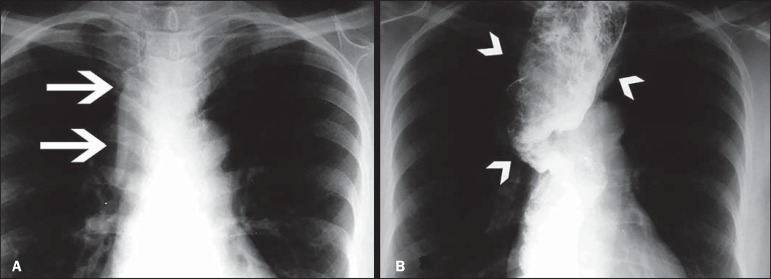
Grade IV megaesophagus. **A**: Posteroanterior X-ray showing widening of the superior mediastinum (arrows). **B**: Barium swallow examination demonstrating a dilated and tortuous esophagus (arrowheads).

**Figure 4:**
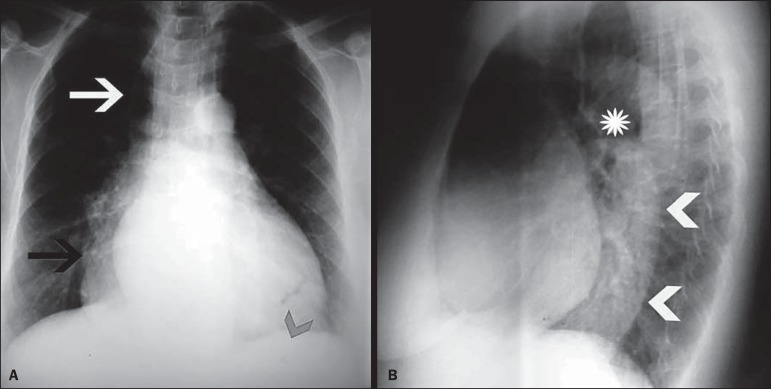
Grade III megaesophagus. **A**: Posteroanterior X-ray showing right inferior mediastinal widening (black arrow) and right superior mediastinal widening, the latter simulating a pneumomediastinum due to gas content inside of the esophagus (white arrow). Absence of the gastric air bubble (arrowhead). **B**: Lateral X-ray showing an air-fluid level (asterisk) and a retrocardiac mass (arrowheads).

The changes found on routine chest X-rays, by Rezende's classification, were as follows (Figures 2, 3, and 4):

- Grade I: None of the patients with grade I esophageal disease showed changes on routine chest X-rays.
- Grade II: On routine chest X-rays, 2 of the 3 patients with grade II esophageal disease presented a change (absence of the gastric air bubble).
- Grade III: Of the 19 patients with grade III esophageal disease, 15 showed changes on routine chest X-rays: absence of the gastric air bubble; presence of an air-fluid level; and changes in the mediastinum, right inferior mediastinal widening being observed in 12 patients, of whom 3 also showed superior widening and 2 also showed left inferior widening.
- Grade IV: All 4 of the patients with grade IV esophageal disease showed changes on routine chest X-rays: absence of the gastric air bubble (in 4); presence of an air-fluid level (in 3); and mediastinal widening (in 4).

## DISCUSSION

Chagas disease can present in acute or chronic form.

The chronic form of the disease can be characterized as involvement of the gastric cardia, the most common being the "mega" syndromes: megacolon and megaesophagus. Gastrointestinal involvement occurs decades after the initial infection with *T. cruzi*. The symptoms related to and morphological changes in the digestive organs occur as a result of alteration and destruction of neurons and nerve ganglia^(5)^.

Megaesophagus is the most common cause of symptoms in patients with the chronic gastrointestinal form of Chagas disease and can occur at any age, although it is most common between 20 and 40 years of age. The speed at which the disease progresses is variable^(6,7)^. In Brazil, Chagas disease is the main cause of achalasia, which affects 7-10% of individuals infected with *T. cruzi* ^(8)^. In patients with Chagas disease-associated megaesophagus, the main presentation is involvement of the submucosal (Meissner) and myenteric (Auerbach) plexuses, impairment of 85% of their neurons having been demonstrated in some cases^(3)^.

The analysis of surgically resected tissue and autopsies of patients with Chagas disease-associated megaesophagus has shown varying degrees of dilation and thickening of the muscle layer, especially of the circular musculature.

In cases of pronounced dilation, such thickening is less apparent and the wall of the esophagus has an atrophic appearance. Microscopic examination of such tissues can reveal local infiltration by lymphocytes, macrophages, and plasmacytes, although the parasite is rarely identified, as well as the loss of neurons from the submucosal (Meissner) and myenteric (Auerbach) plexuses of the esophagus(3,9).

The symptoms of Chagas disease-associated megaesophagus are indistinguishable from those of idiopathic achalasia and include dysphagia, a feeling of fullness after eating or drinking, chest pain, and regurgitation^(9)^. In advanced cases, common complications are bronchial aspiration, weight loss, and cachexia. Hypertrophy of the salivary glands, secondary to hypersalivation, is also seen.

On routine chest X-rays and barium swallow examinations, the appearance of megaesophagus is quite similar to that of achalasia^(9,10)^. In both entities, the esophagus can present density in its vertical soft tissues, located along its right paramediastinal border, in frontal views^(6,7,9,11)^. In some cases, an air-fluid level or food residue can be observed within the esophagus. A common finding in the upper abdomen is a reduced or absent gastric air bubble, due to the restricted air passage through the area of esophageal achalasia^(9)^.

Megaesophagus can be classified in several ways. In the present study, we employed Rezende's classification, which stratifies esophageal involvement into four grades^(4,11)^, as determined by the degree of dilation and changes in esophageal motility.

The diagnosis of Chagas disease-associated megaesophagus can be made by thorough anamnesis, identifying the cause and symptoms suggestive of the disease, together with serologic tests, the "Machado Guerreiro" test battery, chest X-ray, and a barium swallow examination (in real time or filmed for subsequent analysis). The changes found in the barium swallow examination, especially when analyzed in motion (during real-time fluoroscopy or on film), allow the visualization of major changes in the esophagus, such as motility disorders, tertiary waves, delayed emptying, altered caliber, air-barium levels, air-fluid levels, and the "bird beak" sign, which is a conical, symmetrical tapering of the contrast.

In the present study, most of the patients (65.7%) were categorized by barium swallow examination as having advanced esophageal involvement (Rezende grade III or IV). Tertiary waves were identified in all of the patients except in those categorized as having grade IV involvement.

Chest X-rays showed no changes in any of the 9 patients categorized as having grade I involvement, and the only change observed among the patients with grade II involvement was the absence of the gastric air bubble, in 2 (66.6%) of the 3. Of the 19 patients categorized as having grade III involvement, 15 (78.9%) showed changes on routine X-rays, such as absence of the gastric air bubble, in 10 patients (52.6%), an air-fluid level, in 7 (36.8%), and mediastinal widening, in all 15 (78.9%). All 4 of the patients categorized as having grade IV involvement showed the absence of the gastric air bubble and mediastinal widening on routine X-rays. Two of those patients showed an air-fluid level, attributed to esophageal dilation caused by narrowing of the cardia.

Chagas disease-associated megaesophagus can reach quite large dimensions, changing the morphology of mediastinal structures, and can be identified on routine chest X-rays^(9)^. These changes become more common as the disease progresses and are almost exclusive to patients with grade III or IV involvement. Abnormalities such as the absence of the gastric air bubble, the presence of an air-fluid level, and mediastinal widening have been previously reported^(12)^.

A routine chest X-ray alone can raise the suspicion of megaesophagus, which, together with a clinical history suggestive of Chagas disease, can lead to the diagnostic hypothesis of Chagas disease-associated esophageal involvement. Given the small number of patients in our sample, we can illustrate the radiological changes typical of megaesophagus only from a demonstrative (rather than statistical) point of view.

## CONCLUSION

We can conclude that the use of Rezende's classification is feasible. Subtle findings characterizing the early stages of esophageal involvement were found, as was the complete akinesis occurring in cases of severe (sigmoid) megaesophagus, in which the esophagus appears to rest on the right hemidiaphragm because of its voluminous dilation and hypotonia. These chest X-ray findings are more common in patients in the more advanced stages of the disease. Therefore, we can suspect megaesophagus in patients with a clinical and epidemiological history suggestive of Chagas disease. Routine chest X-rays can allow the staging of cases by Rezende's classification, after which the patients can be referred for a more complete and specific assessment in order to diagnose Chagas disease-associated esophageal involvement and can be followed in the most appropriate manner possible.
